# Intraoperative cardiac function assessment by transesophageal echocardiography versus FloTrac/Vigileo™ system during pectus excavatum surgical repair

**DOI:** 10.1186/s44158-021-00025-4

**Published:** 2021-12-09

**Authors:** Silvia Fiorelli, Gelsomina Capua, Cecilia Menna, Claudio Andreetti, Elisabetta Giorni, Ettore Riva, Elisabetta Agostini, Fabrizio D’Andrea, Elisa Massullo, Valentina Peritore, Monica Rocco, Domenico Massullo

**Affiliations:** 1 Anesthesia and Intensive Care Medicine, Department of Clinical and Surgical Translational Medicine, Sapienza University of Rome, Via di Grottarossa, 1035 00189 Roma, Italy; 2 Thoracic Surgery, Department of Clinical and Surgical Translational Medicine, Sapienza University of Rome, Via di Grottarossa, 1035 00189 Rome, Italy; 3 Division of Cardiology, Department of Clinical and Molecular Medicine, University of Rome, Via di Grottarossa, 1035 00189 Rome, Italy

**Keywords:** Pectus excavatum, Cardiac output, Transesophageal echocardiography, Pulse contour analyses, FloTrac/Vigileo

## Abstract

**Background:**

Pectus excavatum (PE), a congenital deformity of the chest wall, can lead to cardiac compression and related symptoms. PE surgical repair can improve cardiac function. Intraoperative transesophageal echocardiography (TEE) has been successfully employed to assess intraoperative hemodynamic variations in patients undergoing PE repair. FloTrac/Vigileo™ system (Edwards Life-sciences Irvine, CA) (FT/V) is a minimally invasive cardiac output monitoring system. This retrospective study aimed to assess hemodynamic changes in surgical repair of PE using FT/V and concordance with parameters measured by TEE.

**Results:**

*N*=19 patients submitted to PE repair via Ravitch or Nuss technique were enrolled. Intraoperative cardiac assessments simultaneously obtained via TEE and FT/V system were investigated. The agreement between TEE-derived cardiac output (CO-TEE) and FT/V system parameter (COAP) was evaluated. The relationship between COTEE and COAP was analyzed for all data using linear regression analysis. A significant correlation between COAP and COTEE values (*R* = 0.65, *p* < 0.001) was found. Bland-Altman analysis of COAP and COTEE showed a bias of 0.13 L/min and a limit of agreement of − 2.33 to 2.58 L/min, with a percentage error of 48%. Intraoperative measurements by TEE and FT/V both showed a significant increase in CO after surgical correction of PE (*p* < 0.005).

**Conclusions:**

FT/V system compared to TEE in hemodynamic monitoring during PE surgery yielded clinically unacceptable results due to a high percentage error. After surgical correction of PE, CO, measured by TEE and FT/V, significantly improved.

## Introduction

Pectus excavatum (PE) is a congenital abnormality characterized by a depression of the anterior chest wall as a result of dorsal deviation of the sternum and of the third to seventh rib or costal cartilage. Pectus excavatum affects approximately 1 in 400 children. Males are afflicted approximately four times more often than females [1]. The two main surgical techniques for PE correction are the modified Ravitch procedure and the Nuss procedure [2], also called minimally invasive repair of PE (MIRPE). The etiology of PE remains unknown. Currently, the underlying pathogenesis of PE is thought to involve overgrowth in the costochondral region of the ribs [3]. Clinical presentation of PE is various: patients can be asymptomatic or affected by exercise limitations or by pain. Most patients are worried about their physical appearance and undergo surgery for esthetic reasons [4].

The cardiopulmonary consequences of these deformities have been widely debated. Specifically, PE is characterized by a reduction of the sterno-vertebral distance and a leftward displacement and rotation of the heart with compression of the right chambers, thus resulting in limitation of diastolic filling and stroke volume [5, 6]. Patients can have documented cardiac compression [7, 8], and almost 80% can show related symptoms [9]. PE surgical repair showed in both adult and pediatric patients an improvement in exercise cardiopulmonary function and exercise tolerance [10–12].

Intraoperative transesophageal echocardiography (TEE) has been successfully employed to assess cardiac function improvements in patients undergoing PE repair [13–15]. TEE allows an accurate analysis, overcoming transthoracic echocardiography limitations due to the abnormal anatomy of the deformed anterior chest wall. TEE requires an adequately trained operator.

FloTrac/Vigileo™ system (Edwards Life-sciences Irvine, CA) (FT/V) is a minimally invasive cardiac output monitoring system useful in a wide variety of medical situations and allows continuous assessment of cardiac output (CO), stroke volume (SV), and stroke volume variation (SVV). At present, no data regarding the use of intraoperative monitoring via fourth-generation FloTrac/Vigileo™ system (FT/V) to assess hemodynamic changes in surgical repair of PE have been reported.

The first aim of this study was to compare intraoperative cardiac output (CO) measurements acquired using the FT/V system by arterial pressure waveform (COAP) and CO measured by transesophageal echocardiography (COTEE) during PE surgery repair. Secondary aims were measurements and assessment of CO changes resulting from the surgical repair.

## Methods

This retrospective study, was approved by the Bioethics Committee of Sapienza University of Rome (no. 6181_2020). All patients submitted to PE repair via modified Ravitch or Nuss technique from January to October 2020 were included in the study. Demographic data, medical history, comorbidities, Haller Index (derived from dividing the transverse diameter of the chest by the anterior-posterior diameter on a simple computerized tomography scan) [16], operative time, surgical approach (Ravitch or Nuss technique), and intraoperative data were collected from the electronic medical record system.

As a standard in this institution, patients received an intraoperative cardiac assessment simultaneously with TEE and minimally invasive monitoring via the FT/V system. Written informed consent was obtained from the patients.

Before anesthesia induction, a 20-G cannula was inserted into the radial artery and then connected to the FloTrac pressure transducer that preprocesses and sends a signal to both the cardiovascular monitor (for real-time waveform display) and to the Vigileo monitor. The FT/V algorithm of hemodynamic calculation has been described in detail [17]. This system samples the arterial waveform at 100 times per second (100 Hz) and calculates pressure wave standard deviation (SD) over a 20-s interval. The system calculates the arterial pressure using arterial pulsatility, resistance, and patient-specific vascular compliance determined from an internal demographic data base (age, sex, height, and weight) and mean arterial pressure using a conversion factor “*χ*.” This factor corresponds to the vascular tone and is calculated through a multivariate polynomial function including pulse rate, body surface area, aortic compliance, mean arterial pressure, skewness, and kurtosis of the arterial pressure. CO was recorded by FT/V before and after PE surgical correction.

The transesophageal probe was inserted after tracheal intubation and the cardiac examination was conducted by an expert operator with an Esaote TM MyLabTM 30 GOLD—CardioVascular (Esaote Italia, Firenze, Italia) before and after surgical correction of the deformity. The preoperative and postoperative TEE measurements were made in apnea by disconnecting the patient from the ventilator. TEE parameters were analyzed and recorded before and after PE surgical correction: right ventricular outflow tract distal diameter (RVOT), left ventricular outflow tract diameter (LVOT), velocity-time integral (VTI) of left ventricular outflow tract systolic flow. All measurements were made according to the recommendations for echocardiographic quantification published by the American Society of Echocardiography [18]:

RVOT: This parameter was measured in midesophageal right ventricular inflow outflow view, transducer angle 20° to 70°. The RVOT diameter was measured 0.5 to 1.0 cm under pulmonary valve at end-diastole and end-systole.

LVOT: This parameter was measured in midesophageal left ventricular outflow tract view, transducer angle 120° to 140°, as the diameter of the left ventricular outflow tract 0.5-1 cm under aortic valve in systole.

LVOT VTI: Pulsed Doppler TEE was performed using a deep trans-gastric approach in order to obtain a deep long-axis view of the left ventricle and placing the sample volume just under the aortic valve, taking care to obtain a smooth Doppler profile, calculating the velocity time integral of the Doppler signal directed across LVOT.

The LVOT area was calculated from the diameter assuming a circular geometry. LVOT VTI was used to estimate SV as it corresponds to the column of blood that moves through the LV outflow tract during each systole. Cardiac output was calculated from the left ventricular outflow tract area, the velocity-time integral of the blood flow profile, and heart rate.

Stroke Volume (SV) = LVOT VTI × cross-sectional area of the left ventricular outflow tract

CO: SV multiplied by heart rate.

### Statistical analysis

All results were expressed as means ± SD. The relationship between COTEE and COAP was analyzed for all data using linear regression analysis. The agreement between COTEE and COAP values was assessed by Bland-Altman analysis [19]. Bias (mean difference between COTEE and COAP) represents the systemic error between the 2 methods. Precision (SD of the bias) represents the random error or variability between the different techniques. The limits of agreement, calculated as bias ±2 SD, define the range in which 95% of the differences between the methods are expected to lie. The percentage error was calculated as the ratio of 2 SD of the bias to the mean CO; this value was considered clinically acceptable if below 30% [20]. The *t* test for paired observation was used to compare the measured values in each patient before and after the surgical correction. *P* values < 0.05 were considered significant. Data were analyzed using the SPSS v25.0 software (SPSS Inc., Chicago, IL, USA).

## Results

Nineteen patients (4 women and 15 men) were included in the study. 38 acceptable pairs of measurements of cardiac output were analyzed: a pre-correction and a post-repair measurement for each patient obtained via TEE and FT/V. Preoperative reported electrocardiography alterations were: right bundle branch block in four patients, incomplete right bundle branch block in five patients, right atrial enlargement in one patient, left axis deviation in one patient, right axis deviation in three patients, left posterior fascicular block in two patients and left ventricular hypertrophy in one patient. No patient was affected by valvular disease. Patients received a moderate intravenous fluid administration (5–6 mL/kg/h), and no significant variations in fluid balance were reported.

The demographic data are represented in Table 1.

**Table 1:** Demographic and perioperative data of the enrolled population

Parameter	
**Gender (M/F)**	15/4
**Age (years)**	23.63 ±7.19
**Height (cm)**	180.68±7.52
**Weight (kg)**	66.58±12.06
**BMI (kg/m**^**2**^**)**	20.19±2.40
**ASA (I/II)**	11/8
**Preoperative EF (%)**	63.75±2.49
**Preoperative METs**	9.79±0.53
**Preoperative ECG alterations:**	
**RBBB**	4
**IRBB**	5
**LPFB**	2
**RAE**	1
**LAD**	1
**RAD**	3
**LVH**	1
**Surgery (Ravitch/Nuss)**	12/7
**Haller’s index**	3.47±0.42
**Anesthesia duration (min)**	112.78±44.44
**Surgery duration (min)**	86.11±39.19

The data is expressed as mean ± SD or N° of patients

*ASA* American Society of Anesthesiologists, *BMI* Body mass index, *F* Female, *M* Male, *ECG* Electrocardiography, *EF* Ejection fraction, *METs* Metabolic equivalents, *RBBB* Right bundle branch block, *IRBBB* Incomplete right bundle branch block, *RAE* Right atrial enlargement, *LAD* Left axis deviation, *RAD* Right axis deviation, *LPFB* Left posterior fascicular block, *LVH* Left ventricular hypertrophy

**Fig. 1:**
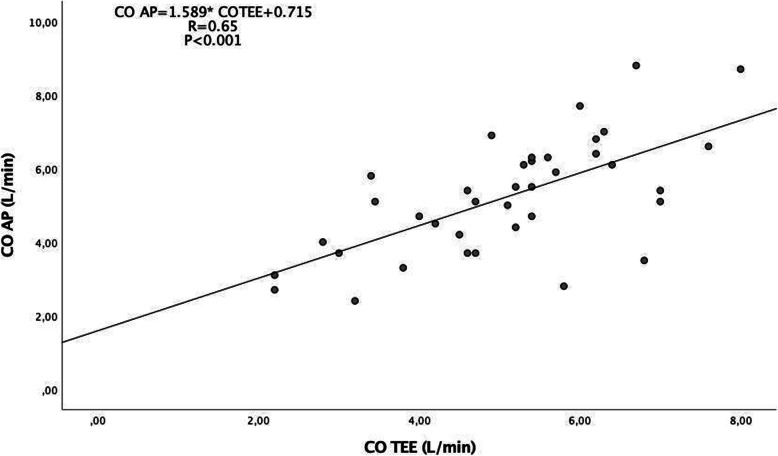
The relationship between COAP and COTEE. There was a significant correlation between COAP and COTEE during pectus excavatum repair surgery (*R* = 0.65, *p* < 0.001). COAP, arterial pressure waveform cardiac output; COTEE, transesophageal echocardiography measured cardiac output

**Fig. 2:**
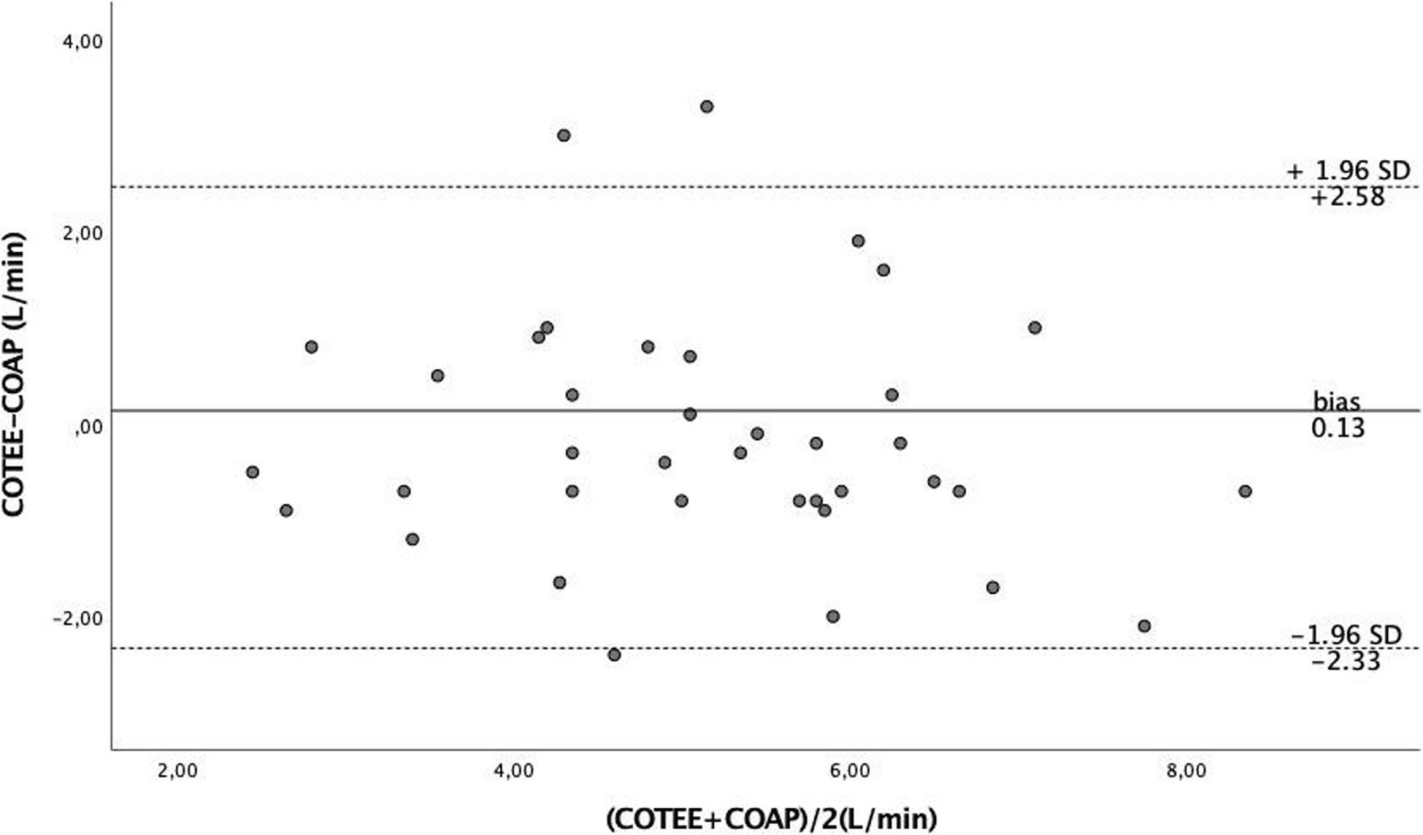
Bland-Altman plot between COAP and COTEE. Bias was 0.13, and limits of agreement were 2.58 and − 2.33. COAP, arterial pressure waveform cardiac output; COTEE, transesophageal echocardiography measured cardiac output

Analysis of the overall relationship between COTEE and COAP (Fig. 1) showed a significant correlation between COTEE and COAP (*R* = 0.65, *p* < 0.001). The Bland-Altman plot displays the limits of agreement between COTEE and COAP (Fig. 2), revealing a bias of 0.13 L/min and a limit of agreement of − 2.33 to 2.58 L/min. The percentage error of all data was 48%. Comparisons of CO determinations were made are shown in Table 2.

**Table 2:** Cardiac output (CO) intraoperative measurements

		BIAS (L/min)	Precision	95% Limit of Agreement (L/min)	Percentage Error
**CO**	**Before correction**	0.25	1.18	− 2.06 to 2.56	51
	**After correction**	− 0.15	1.34	− 2.77 to 2.47	46

*CO* Cardiac output

The mean values of the COTEE and COAP before and after surgical correction of PE are summarized in Table 3. The mean values of RVOT before and after correction were 20.21±5.15 and 24.73±4.05, *p*=0.001.

**Table 3:** Cardiac output (CO) assessed by intraoperative transesophageal echocardiography (COTEE) and using the Flotrac/Vigileo system by arterial pressure waveform (COAP) before and after surgical correction of pectus excavatum deformity

Intraoperative data	Before correction	After correction	Intraoperative variation (Δ)	***P*** value*
**COTEE**	4.47±1.32^†^	5.73±1.27^‡^	1.25±1.03^$^	**0.001**
**COAP**	4.72±1.54	5.75±1.45	1.04±1.31	**0.004**

*p value by sample paired *t* test; ^†^*p* = 0.362 vs COAP; ^‡^*P* = 0.960 vs COAP; ^$^*p*=0.514 vs ΔCOAP

## Discussion

The main findings of this study indicate that overall COAP yielded clinically unacceptable results during PE repair surgery, although they showed a significant correlation with COTEE. The mean values of the COTEE and COAP significantly improved after surgical correction of PE.

The “gold standard” method for measuring CO in the clinical setting is considered thermodilution using a pulmonary artery catheter (PAC) [21–23]. However, because the risk of PAC insertion-related risks cannot be justified in non-cardiac surgery, less invasive methods are commonly employed.

Pulse contour devices showed to be less dependable compared to Doppler-derived CO because they miss compensating for circulatory modifications in peripheral resistance. FT/V showed greater bias in both low and high systemic vascular resistance (SVR) states compared to thermodilution [20]. Despite the fourth-generation algorithm can adjust for acute modifications in SVR, its precision, accuracy, and trending ability are still clinically unacceptable [24].

The estimations made with FT/V showed a considerable bias and a wide range of levels of agreement compared to PAC [25–28]. Form analysis of arterial wave algorithms is founded on features of the arterial system, such as impedance, peripheral vascular resistance, and compliance. Aortic impedance is necessary to calculate SV and varies considerably from patient to patient. These interindividual discrepancies in aortic impedance may participate to inaccuracies in calculating the CO when calibration is founded only upon demographic data.

When repeated hemodynamic variations occur, like during surgery for PE repair, the k value may be delineated at a time in which vascular peripheral resistance, arterial compliance, or impedance may not be the same as the moment of estimation [29]. The results of this study, are according to previous reports comparing FT/V and TEE, showing that COAP values measured by the Flotrac/Vigileo system were not clinically acceptable [28–31]. Concha et al. during laparoscopic colon surgery reported considerable variations between CO measurements obtained with TEE and FT/V; bias was 1.17 and limits of agreement − 2.02 and 4.37, and the percentage error was 40% [28]. During surgery for abdominal aortic aneurysm, COAP assessments demonstrated to be not clinically acceptable because of extensive changes during aortic clamping and declamping (bias of 0.12 L/min and limits of agreement 1.66 to 1.90 L/min, with a percentage error of 41%) [30].

Although FT/V has failed to show that it is comparable to TEE for cardiac monitoring during surgical repair of PE, this tool can be helpful for intraoperative management. The use of this minimally invasive system can allow even less experienced personnel to monitor cardiac changes during PE surgical correction. It was also shown that hemodynamic measures vary significantly and almost instantaneously following surgical decompression, and the FT/V system has the advantage to allow continuous monitoring of these parameters. During PE surgery, major complications can occur. They include bleeding due to possible perforation of the heart and large vessels, bleeding of the chest wall, right ventricular compression, hypotension, and arrhythmias, which can be promptly identified and treated thanks to the aid of continuous hemodynamic monitoring [32].

This study has some limitations. Although thermodilution was not used as a gold standard reference because it would require a PAC, which would represent an additional unjustifiable risk in these patients, CO measured by TEE was previously reported as clinically acceptable and is a well-validated tool in reporting hemodynamic changes in PE repair surgery [33]. In addition, COTEE takes some minutes to calculate CO. As a result, CO measurements by FT/V and TEE were not exactly reported at the same timepoint. Another limit is that TEE is an operator-dependent technique. However, when a good determination of the aortic valve area and proper alignment of the ultrasound beam and the LVOT are obtained, there is an agreement between TEE and PAC [33]. An additional possible limitation of the study is that the results could have been conditioned by outlier data because we investigated CO estimations obtained by only 19 patients. Finally, the limit of the retrospective nature of this study could be overcome with future prospective trials.

In conclusion, the FT/V system compared to TEE in hemodynamic monitoring during PE surgery was not clinically acceptable due to a high percentage error. Nevertheless, FT/V was able to monitor and detect an intraoperative increase of hemodynamic parameters after correction.

## Data Availability

The data that support the findings of this study are available upon reasonable request.

## Abbreviations

CO: Cardiac output
COAP: Cardiac output measured by FloTrac/Vigileo™ system by arterial pressure waveform
COTEE: Cardiac output measured by transesophageal echocardiography
FT/V: FloTrac/Vigileo™ system
LVOT: Left ventricular outflow tract diameter
MIRPE: Minimally invasive repair of pectus excavatum
PAC: Pulmonary artery catheter
PE: Pectus excavatum
RVOT: Right ventricular outflow tract distal diameter
SD: Standard deviation
SV: Stroke volume
SVR: Systemic vascular resistance
SVV: Stroke volume variation
TEE: Transesophageal echocardiography
VTI: Velocity-time integral
